# Neural Regeneration in Regenerative Endodontic Treatment: An Overview and Current Trends

**DOI:** 10.3390/ijms232415492

**Published:** 2022-12-07

**Authors:** Yali Wei, Ping Lyu, Ruiye Bi, Xinyu Chen, Yanshen Yu, Zucen Li, Yi Fan

**Affiliations:** 1 State Key Laboratory of Oral Diseases, National Clinical Research Center for Oral Diseases, Department of Cariology and Endodontics, West China Hospital of Stomatology, Sichuan University, Chengdu 610041, China; 2 State Key Laboratory of Oral Diseases, Department of Oral and Maxillofacial Surgery, National Clinical Research Center for Oral Diseases, West China Hospital of Stomatology, Sichuan University, Chengdu 610041, China; 3 State Key Laboratory of Oral Diseases, National Clinical Research Centre for Oral Diseases, West China Hospital of Stomatology, Sichuan University, Chengdu 610041, China

**Keywords:** dental pulp necrosis, tissue engineering, stem cells, growth factors, scaffolds

## Abstract

Pulpal and periapical diseases are the most common dental diseases. The traditional treatment is root canal therapy, which achieves satisfactory therapeutic outcomes—especially for mature permanent teeth. Apexification, pulpotomy, and pulp revascularization are common techniques used for immature permanent teeth to accelerate the development of the root. However, there are obstacles to achieving functional pulp regeneration. Recently, two methods have been proposed based on tissue engineering: stem cell transplantation, and cell homing. One of the goals of functional pulp regeneration is to achieve innervation. Nerves play a vital role in dentin formation, nutrition, sensation, and defense in the pulp. Successful neural regeneration faces tough challenges in both animal studies and clinical trials. Investigation of the regeneration and repair of the nerves in the pulp has become a serious undertaking. In this review, we summarize the current understanding of the key stem cells, signaling molecules, and biomaterials that could promote neural regeneration as part of pulp regeneration. We also discuss the challenges in preclinical or clinical neural regeneration applications to guide deep research in the future.

## 1. Introduction

Dental pulp is the only soft tissue in the tooth. It is located in the dental pulp cavity and is surrounded by dentin and connected to the periapical tissue by the narrow apical foramen [1,2]. It has several functions, including tooth formation, nutrition, sensation, and defense in physiological circumstances. When it is subjected to abnormal external stimuli such as caries or abrasion, it produces a defensive response by forming tertiary dentin to protect the pulp. Dental pulp maintains the vitality of dentin by providing oxygen and nutrients to odontoblasts and cell processes, performing sensory function through rich nerve distribution, and activating immune or inflammatory cells to participate in defense by regulating various molecular mediators [3]. The structure of dental pulp makes it difficult to restore its normal physiological state after bacterial infection or trauma. Root canal therapy (RCT) is the most effective and common treatment for pulpal and periapical diseases. By preparing, disinfecting, and filling root canals, we can control infection and accelerate the healing of periapical lesions. Nevertheless, due to the loss of vital pulp after RCT, the normal function of the pulp cannot be restored, resulting in increased fragility of the tooth, loss of defense ability, and even extraction [4].

The term “Regenerative endodontic therapy (RET)” was first proposed by Murray et al. [5] in 2007 based on tissue engineering. RET was conceived to replace inflamed/necrotic pulp tissue by regenerating pulp-like tissues [6]. At present, the most commonly used RET in clinical practice is revascularization. Revascularization is a method of recruiting undifferentiated oral stem cells and molecules from the apical region using manual files to guide a bleeding clot (BC), or using platelet-rich plasma (PRP) or platelet-rich fibrin (PRF) after adequate disinfection of the root canal in order to promote the development of immature permanent teeth. Although various studies have proven the efficacy of revascularization, many scholars suggest that revascularization may not achieve real pulp regeneration [7]. Therefore, two new strategies based on tissue engineering have been proposed: stem cell transplantation, and cell homing. Stem cell transplantation entails transplanting stem cells combined with growth factors (GFs) and scaffolds into the root canal. Further formation of a new pulp–dentin complex is accomplished by promoting stem cells’ differentiation towards odontoblasts [8]. Dental stem cells—such as dental pulp stem cells (DPSCs), stem cells from apical papilla (SCAPs), human exfoliated deciduous teeth (SHEDs), and periodontal ligament stem cells (PDLSCs)—are widely applied for stem cell transplantation owing to their excellent capability for multiple differentiation [9]. Cell homing aims to implant GFs with scaffolds into the root canal to build an environment conducive for tissue regeneration. Then, autologous stem cells are recruited from the periapical region and into the root canal to realize RET [10]. The most widely used materials for scaffolds, whether in cell transplantation or cell homing, are natural materials such as collagen [11].

The ideal pulp regeneration process should not only form the same structure as the natural dental pulp but also restore its function. Functional dental pulp should meet the following requirements [12]: (1) deposition of new dentin at an adjustable rate; (2) cell density and structure similar to those of natural dental pulp; (3) vascularization; and (4) innervation. Normal pulp is rich in nerve fibers. The number of nerve fibers in the regenerated pulp determines the degree of restoration of sensory function. Therefore, nerves are indispensable in regenerating dental pulp, and particularly important for maintaining its structure and function. This review summarizes the recent studies of successful neural regeneration in RET in vivo, analyzes the conditions and requirements for achieving neural regeneration, and discusses stem cells, GFs, and scaffolds that can promote neural regeneration. We believe that this review can provide a reference for clinical translation in guiding neural regeneration in RET in the future.

## 2. Function of Nerve in Dental Pulp

The nerves of the pulp are mainly derived from the maxillary and mandibular branches of the trigeminus. The sensory nerve fibers enter the pulp cavity via blood vessels through the apical foramen. The nerve fibers of the pulp are mixed fibers, since they also contain sympathetic fibers from cervical sympathetic nodes [13]. The sensory nerve fibers of pulp include myelinated Aδ fibers and unmyelinated C fibers. These are both responsible for transmitting pain impulses [14]. Because there are only nociceptors in the pulp, pain results when the pulp is stimulated by mechanical or temperature changes. The terminals of Aδ fibers are distributed in the pulp–dentin junction area, have a low stimulation threshold, and produce pain characterized by acute tingling. The terminals of the C fibers are spread throughout the pulp and have a high stimulation threshold; thus, the pain is characterized by a severe burning-like sensation [15]. The stimulation of C fibers is closely related to the pain caused by inflammation. When the pulp is inflamed, local tissue pressure increases, which excites the C fibers and transmits the sense of pain. On the other hand, inflammatory mediators such as histamine and bradykinin can excite C fibers, which also release neuropeptides such as substance P (SP) and calcitonin-gene-related peptides (CGRPs). This results in vasodilation and increased peripheral nerve sensitivity [16]. C fibers are resistant to hypoxic environments, and patients may still feel pain when the pulp is hypoxic and necrosis [17].

**Figure 1:**
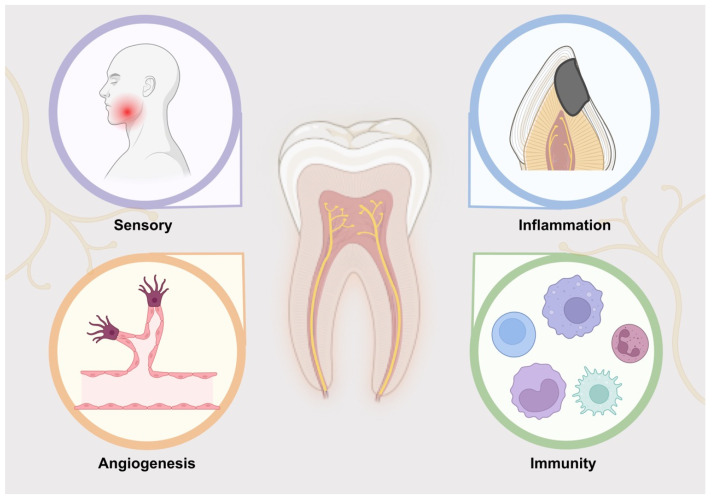
Nerve function in teeth. Nerves participate in sensation, inflammation, angiogenesis, and immunity in the tooth.

Sensory neurons also participate in the regulation of inflammation, immunity, and angiogenesis, mainly through the secretion of nerve growth factor (NGF) and neuropeptides (Figure 1). In dental caries, fibroblasts located directly below the caries secrete NGF to promote axon growth to the damaged area [18]. The newborn nerve terminals form processes in the pulp angle area in response to the damage of dental caries. In pulpitis, NGF is continuously upregulated to increase the density of nerve fibers [14]. Moreover, C fibers and non-nerve cells secrete neuropeptides to participate in the transmission of pain, and the level of neuropeptides significantly increases with inflammation. Furthermore, various resident cells in the pulp—such as fibroblasts, odontoblasts, inflammatory cells, and endothelial cells of the postcapillary venules—express membrane-binding receptors of sensory and sympathetic peptides [19]. During inflammation, the expression of these receptors increases significantly, indicating that neuropeptides are crucial in mediating inflammation [19]. The nerve in the pulp also participates in vascular regulation. The sympathetic autonomic nerve of the superior cervical ganglion affects the contraction of blood vessels in the pulp [15]. Moreover, the release of CGRP and SP promotes vasodilation and plasma extravasation [20]. Regarding immune system regulation, a study demonstrated that the recruitment of immunoreactive cells in the inflammatory pulp of denervated molars was significantly lower than that of innervated molars, implying that nerves are involved in the immune system processes of pulp [21]. Ho et al. [22] found that many receptors of neuronal signal molecules existed in immune cells, such as monocytes/macrophages, indicating that nerves were directly involved in the recruitment of immune cells.

## 3. Development of Neural Regeneration in RET

Pulp is a well-vascularized neurological tissue. After years of research and discussion, most in vivo RET experiments and clinical trials have successfully achieved the regeneration of pulp-like tissue with vessels. However, nerve formation is still problematic, and few experiments have reported nerve formation in RET. To truly achieve functional pulp regeneration, restoring innervation is indispensable. 

Revascularization is the method most commonly used to promote the continuous development of roots and the closure of the apical foramen of immature permanent teeth. However, histological analysis shows that the regenerated tissue is not pulp-like, but more periodontal-like, similar to cementum. Most of the roots develop after revascularization and are positive to sensitivity tests, which are helpful to guide neurogenesis in functional pulp regeneration. A study indicated that vitality tests required periapical neuronal terminals to be located in the coronal potion of the repaired tissue, about 10-15 mm from the nearest nerve trunk [23]. Another study analyzed the tooth #45 of a 10-year-old child who underwent revascularization. The results showed that ectopic cementum and periodontal-ligament-like tissue were located in the root canal, while nerve fibers were found by immunohistochemistry [24]. Furthermore, Austah et al. [25] reported that they successfully promoted root development of necrotic pulp by revascularization and that the teeth were positive to sensitivity tests. Immunohistochemistry further indicated that there were CGRP-positive nerve fibers. These experiments further confirmed that the reconstruction of sensory fibers was successfully induced by revascularization. 

During human tooth development, the innervation of the teeth is modulated by a series of molecular signals that are mainly regulated by the release of neurotrophic factors (NFs)—especially NGF and brain-derived neurotrophic factor (BDNF) [26]. The primary afferent branches accumulate around the apical papilla and then enter the dental pulp until the late cap stage to form the peripheral primary afferent branches of the pulp–dentin complex [23,27]. Therefore, it is necessary to provide the appropriate concentration of NFs for the growth of nerve fibers in RET. In addition to the exogenous provision of NFs, some mesenchymal stem cells (MSCs)—such as DPSCs, SCAPs, and SHEDs—can secrete NFs. Moreover, various studies have confirmed that dental MSCs not only have nerve differentiation potential but also secrete other molecules such as chemokines and vascular GFs, which can regulate axon growth and accelerate the recovery of nerves [28]. The reinnervation of dental pulp needs to be guided by axons to recruit free nerve terminals in the apical area. Therefore, finding suitable stem cells, NFs, and related scaffolds is particularly important.

Revascularization requires comprehensive disinfection of the root canal. The root canal can be continuously irrigated with sodium hypochlorite during the first treatment, and then calcium hydroxide or antibiotic paste can be injected into the root canal for disinfection. If there are no signs of infection during the revisit, dry the canal after removing the disinfectant and use a sterile K-file beyond the apical foramen to induce apical bleeding and fill the root canal with blood. PRP or PRF can be used instead of this step. After the blood clot in the root canal has formed, cover it with a layer of mineral trioxide aggregate (MTA)/Biodentine or other materials. The defect can then be filled with glass ionomer cement. Patients need follow-up for a long time after treatment. Stem cell transplantation and cell homing also require root canal preparation and adequate disinfection. Hydrogel scaffold materials such as collagen, which is the most widely used, can be loaded with stem cells such as DPSCs, SHEDs, bone marrow mesenchymal stem cells (BMMSCs), or adipose-derived stem cells (ADSCs), and can be injected into the canals with or without GFs, such as platelet-derived growth factor (PDGF)-BB, NGF, BDNF, granulocyte colony-stimulating factor (G-CSF), stromal-cell-derived factor-1 (SDF-1), basic fibroblast growth factor (bFGF), or bone morphogenetic protein 7 (BMP7). Regular follow-up is also necessary.

We summarized the recent successful reports of neural regeneration in animal experiments and clinical trials of pulp regeneration, as shown in Table 1 and Table 2. Our certification of successful neuroregenerative experiments included observing nerves through histological analysis or confirming classical nerve markers by immunofluorescence. Furthermore, we considered positivity for sensitivity tests, including cold tests, hot tests, and electric pulp tests (EPTs).

## 4. Role of Stem Cells in Neural Regeneration

As key components of tissue engineering, stem cells are essential in pulp regeneration. Stem cells commonly used for RET can be classified as dental and non-dental cells. Dental stem cells include DPSCs, SHEDs, PDLSCs, SCAPs, and dental follicle stem cells (DFSCs) [89,90,91]. Non-dental stem cells mainly include BMMSCs, ADSCs, embryonic stem cells (ESCs), and induced pluripotent stem cells (iPSCs) [92,93]. Dental stem cells are the favored seed cells for pulp regeneration because they are non-invasive, have excellent proliferative activity, and possess neural and vascular differentiation ability. Compared with ESCs, dental stem cells pose fewer ethical problems. Non-dental stem cells also exhibit potential in pulp regeneration. Stem cells with neural differentiation characteristics are discussed in this session.

### 4.1. DPSCs

DPSCs are obtained from mature pulp tissue and have high colony-formation and proliferation rates. They are regarded as the “gold standard” for stem cells in dental tissue engineering. Gronthos et al. [94] first identified and isolated human DPSCs from human third molars. They can be obtained mainly from the third molar or orthodontic teeth that need to be extracted. DPSCs are MSCs derived from the ectoderm and originate from the cranial crest during embryonic development. They express not only MSC markers such as CD10, CD13, CD29, CD44, CD73, CD90, CD105, CD106, CD117, CD146, and STRO-1, but also neural crest markers such as p75, Snail-1 and -2, and Sry-type high-mobility-group box 9 (SOX-9) [95]. In addition, some multipotent stem cell markers—such as octamer-binding transcription factor 4 (Oct4), Nanog, SOX-2, stage-specific embryonic antigens (SSEAs), and c-Myc—are also expressed in these cells [96]. In vitro, the differentiation of DPSCs is regulated by the microenvironment. DPSCs have the potential to differentiate into multiple cell types—for example, osteoblasts, muscle cells, adipose cells, melanoma cells, chondrocytes, and corneal epithelial cells [91,97]. A lineage-tracing experiment found that peripheral-nerve-associated glial cells differentiate into dental MSCs during the development, self-renewal, and repair of teeth [98]. This confirmed the neurological origin of dental MSCs. DPSCs can differentiate into glial cells and neurons and can secrete NFs, including BDNF, GDNF, NGF, and neurotrophin-3 (NT-3) [92]. Gronthos et al. [99] explored the neural markers of DPSCs for the first time. The results were positive for neuroepithelial stem cell protein (Nestin) and glial fibrillary acidic protein (GFAP). Moreover, dental pulp cell sheets produced neurotrophins, which promoted neural regeneration and enhanced the function of an injured facial nerve in rats [100]. In addition, DPSCs protected and accelerated the regeneration of retinal ganglion cells in vivo and in vitro [101,102]. Furthermore, Kolar et al. [103] reported that axon growth inhibitors existed in completely transected spinal cord injury (SCI), but the nutritional factors secreted by human DPSCs promoted axon regeneration. The mechanism that DPSCs use to accelerate the repair and regeneration of multiple neurological diseases may be viewed from the following four perspectives: differentiated into neurological cells, paracrine, anti-inflammatory, and immunomodulatory [104].

An experiment recorded the positive staining for nerves in pulp-like tissue regenerated by DPSCs [105]. In addition, Iohara et al. [39] regenerated pulp-like tissue with dentin and dense nerve plexus in the pulp cavity of a beagle dog’s permanent teeth with collagen loaded with DPSCs and GCS-F, confirming the role of DPSCs in neural regeneration. Overall, these findings suggest that DPSC transplantation promotes axon extension and neuronal growth in pulp regeneration [8]. A CD31^-^/CD146^-^ population in DPSCs found with high expression of neurotrophic and angiotrophic factors is expected to become the best candidate of stem cells for functional pulp regeneration [106].

### 4.2. SHEDs

SHEDs are extracted from the pulp of human deciduous primary teeth. They are similar to DPSCs and are considered to be a precious *stem cell source* in RET [107]. Miura et al. [108] discovered SHEDs in 2003. They showed better proliferation and self-renewal ability than DPSCs and BMMSCs. Similarly, markers of MSCs such as CD13, CD29, CD44, CD73, CD90, CD105, CD106, CD146, CD166, and STRO-1 are detected on the surface of SHEDs. However, hematopoietic stem cell markers such as CD14, CD18, CD19, CD24, CD34, and CD45 are negative [109]. SHEDs also have multiple differentiation potentials, such as osteogenesis, dentinogenesis, adipogenesis, and neurogenesis. Additionally, the osteogenic and adipogenic differentiation ability of SHEDs is more potent than that of DPSCs [110]. Because of the neural origin of pulp cells, SHEDs also express neuronal markers such as Nestin, NeuN, GFAP, Doublecortin, β III-tubulin, and glutamic acid decarboxylase (GAD) [107]. Studies have demonstrated that SHEDs form neurospheres in the culture medium and then differentiate into dopaminergic neuron clusters [111]. Additionally, SHEDs can also differentiate into Schwann cells (SCs) and accelerate axon regeneration [112]. It has been observed that SHEDs cultured in vitro produce NFs, such as NGF, BDNF, and GDNF [112]. An in vitro study reported that SHEDs expressed more neuronal markers than DPSCs and had a superior ability to differentiate into neurocytes [113]. 

In pulp regeneration, SHEDs and hydroxyapatite/tricalcium phosphate (HA/TCP) were combined for the first time in 2003 and demonstrated that dentin was formed in the scaffold [114]. Rosa et al. [115] implanted SHEDs with biological scaffolds (i.e., Puramatrix^TM^ or rhCollagen type I) into full-length root canals, which were transplanted subcutaneously into the dorsal surface of immunocompromised mice. They found that SHEDs differentiated into odontoblasts with secretory functions and formed functional dental pulp and tubular dentin. In a randomized clinical trial, autologous SHED transplantation regenerated vascularized pulp-like tissue with sensory nerves in traumatic teeth in children [34]. As adult stem cells, SHEDs have unique advantages. They are derived from naturally replaced deciduous pulp; thus, they can be obtained non-invasively and involve less ethical controversy. Moreover, they can effectively avoid immune rejection when used for autografting [92,110]. 

### 4.3. PDLSCs

PDLSCs are found in the periodontal ligament (PDL) between the teeth and the alveolar fossa, where they maintain the homeostasis of the periodontal tissue. PDLSCs were first isolated from periodontal ligaments by Seo et al. in 2004 [116]. They are heterogeneous, clonal, and highly proliferative multipotent cells that produce collagen and PDL-like tissue to promote the regeneration of periodontal tissue under periodontal diseases [117]. PDLSCs can differentiate into osteoblasts, chondrocytes, adipocytes, cardiomyocytes, endothelial cells, islet cells, and corneal keratinocytes [118]. They express typical MSC markers such as CD10, CD13, CD26, CD29, CD44, CD59, CD73, CD90, CD105, CD106, CD140b, CD146, and CD166 [109]. In addition, PDLSCs can express neuron markers, glial cell markers, and early markers of neural stem cells (e.g., human natural killer 1, p75, Nestin), indicating their great potential in the repair of nervous system injury [118,119]. A study found that PDLSCs transplanted into the brains of adult mice survive, migrate, and differentiate into neuron-like cells [120]. Moreover, Fortino et al. [121] used epidermal growth factor (EGF) combined with bFGF to induce PDLSCs to differentiate into neuron cells. 

PDLSCs are prone to forming directional fibers and can differentiate into glial cells and osteoblasts, but their neural differentiation ability is poorer than that of other dental stem cells [122]. However, PDLSCs mainly form PDL-like or cementum tissue in vivo, and related research on their use in RET is still in its infancy. Thus, their potential needs to be investigated further.

### 4.4. SCAPs

SCAPs come from the immature root apical region of permanent teeth and share the same origin as DPSCs [123,124]. Although apical papilla is a precursor tissue of root pulp, there are still significant differences in the biological characteristics of DPSCs and SCAPs [125]. Some scholars believe that SCAPs differentiate into primary odontoblasts when root dentin is formed during tooth development, while DPSCs may be the source for replacement odontoblasts to form reparative dentin [122]. Compared with DPSCs, SCAPs exhibit higher proliferation and tooth tissue regeneration ability. This may be related to the high expression of survivin and telomerase in SCAPs [126,127]. SCAPs express many odontogenic markers, but the expression levels of dentin sialophosphoprotein (DSPP), matrix extracellular phosphoglycoprotein (MEPE), transforming growth factor β RII, FGFR3, VEGFR-1, FGFR1, and CD146 in SCAPs are lower than those in DPSCs [128]. In addition, SCAPs not only express classical MSC markers but also have a SCAP-specific marker—CD24 [129]. Moreover, they are positive for neuron markers such as β III tubulin, glutamic acid decarboxylase, NeuN, Nestin, neurofilament protein M (NF-M), and neuron-specific enolase (NSE) [126]. SCAPs can differentiate into odontoblasts, osteoblasts, adipocytes, and neural cells in vitro [130]. In addition, the differentiation of SCAPs into functional odontoblasts has been verified in vivo. Research has demonstrated that SCAPs can regenerate a dentin-pulp-like complex in immunodeficient mice with appropriate scaffolds [127]. Moreover, it has been confirmed that the secretion of BDNF from SCAPs is crucial for neuronal growth in vitro [131]. Semi-quantitative RT-PCR confirmed that the gene expression levels of *BDNF*, *GDNF*, and *ANGPT1* in SCAPs were much higher than those in other dental MSCs [103]. This indicates the potential of SCAPs for neural regeneration. 

Sequeira et al. [132] investigated the pulp regeneration potential of SCAPs. SCAPs were embedded in PRP scaffolds and then implanted into the dorsal region of immunodeficient rats. They successfully achieved formation of pulp-like and dentin-like tissue. Since their anatomical location is near the apical foramen, SCAPs are also the most important candidate stem cells for revascularization [133]. However, in vivo analyses using SCAPs are still scarce, and the neural differentiation potential of SCAPs in RET needs further investigation.

### 4.5. BMMSCs

BMMSCs were the first MSCs to be discovered, and bone marrow is a widely used source of MSCs. The relevant characteristics of BMMSCs have been widely studied and applied [134]. The positive markers for BMMSCs are Stro-1, CD271, CD146, CD106, CD73, CD105, FZD9, SUSD2, LEPR, and CD90, and the negative markers are CD44, CD31, CD34, and CD45 [135]. BMMSCs are a subgroup of stromal cells. They can be isolated from compact bone, cancellous bone, and the femoral head [136]. BMMSCs can differentiate into osteoblasts, chondroblasts, adipocytes, myoblasts, and neurons [137,138]. In terms of whole-tooth regeneration, BMMSCs upregulate the expression of tooth-derived genes and recombine with embryonic oral epithelium [139]. Furthermore, C-Kit (+) BMMSCs can differentiate into odontoblasts and periodontal tissue cells [140,141,142]. Specifically, BMMSCs can differentiate into neurons, astrocytes, and SCs [143]. Experiments have also exhibited the neural differentiation potential of BMMSCs in vivo. A study demonstrated that BMMSCs differentiated into neuron-like cells after being transplanted into rats [144]. Mathot et al. [145] used BMMSCs to repair 10 mm sciatic nerve defects in rats. It was found that both differentiated and undifferentiated BMMSCs are able to promote the repair of the injured nerve without significant differences. In addition, another study combined BMMSCs and iRoot BP to explore their effects on pulp-like tissue formation. The results showed that the newborn tissue was pulp-like tissue with positive neural markers [146].

### 4.6. ADSCs

ADSCs are MSCs with multi-differentiation potential. They were first isolated from adipose tissue by Zuk et al. [147] in 2001. They are widely utilized in regenerative medicine and are an excellent substitute for BMMSCs in bone repair and regeneration [148,149]. ADSCs express the surface markers of MSCs, such as CD10, CD13, CD29, CD44, CD49, CD73, CD90, CD105, and CD166, but not CD11b, CD14, CD31, or HLA-DR [150]. ADSCs can protect and promote the regeneration of the central nervous system by secreting BDNF, GDNF, NGF, and insulin-like growth factors (IGFs) [151]. An in vivo study investigated the effect of silicone catheters on the repair of 7 mm facial nerve defects in rats, which contained undifferentiated ADSCs, differentiated ADSCs, or SCs [152]. Morphological quantitative analysis of the regenerated facial nerves demonstrated that undifferentiated ADSCs, differentiated ADSCs, and SCs had similar neural regeneration potential [152].

Ishizaka et al. [40] found that CD31^-^ ADSCs induced pulp regeneration. The qualitative and quantitative patterns of mRNA expression in newborn tissue were similar to those of natural pulp. Moreover, Murakami et al. [36] successfully achieved pulp regeneration with ADSCs and BMMSCs, accompanied by angiogenesis and nerve regeneration. These results confirm the role of ADSCs in RET and neural regeneration. The extraordinary biological characteristics of ADSCs have made them vital seed cells in the field of oral regeneration, but their immunogenicity and safety after transplantation are still a concern. 

## 5. Role of GFs in Neural Regeneration

GFs are crucial in tissue engineering. Different GFs combined with scaffolds promote endogenous tissue regeneration—especially those related to vascular, nerve, and dentin formation. GFs such as PDGF and VEGF are related to angiogenesis. SDF-1 α, bFGF, and PDGF are used for chemotaxis, while BMP-7 is used to promote odontoblast differentiation and mineralization [153,154]. Some GFs promote neuronal growth and provide protection, including NGF, BDGF, NT-3, bFGF, IGF, G-CSF, GDNF, and EGF. The following section summarizes the GFs that promote nerve growth and are widely used in RET.

### 5.1. NGF

NGF was the first neurotrophin found by Rita Levi-Montalcini and Viktor Hamburger [155] in murine sarcoma culture. It is a vital regulator of the fate, development, survival, and growth of neurons and non-neurons [156,157]. It has been demonstrated that NGF has two receptors: TrkA and p75NTR. The affinity between NGF and the transmembrane glycoprotein receptor p75NTR is low, and the activation of p75NTR determines cell survival or apoptosis [158]. NGF has a high affinity for TrkA, but its function is affected by p75NTR [159]. It has been found that TrkA and p75NTR are involved in cell proliferation, differentiation, and survival in bone marrow and lymphoid tissue [160]. Moreover, NGF can promote the differentiation and migration of vascular smooth muscle cells and endothelial cells, mobilize and activate endothelial progenitor cells, and promote neovascularization [161]. It has been reported that NGF induces BMMSCs to differentiate into neural cells via a neuropeptide pathway, verifying the capability of NGF for neural regeneration [162]. Chung et al. [163] found that NGF induced neurogenesis of dopaminergic cells through an ERK-driven and transcription-dependent latent process and an ERK- and PI3K-driven and transcription-independent extension process. Furthermore, NGF, in combination with other NFs, successfully induced DPSCs to differentiate into neurogenic cells [164].

In dentistry, NGF induces SCAPs’ differentiation into odontoblasts. This indicates that NGF can be applied as a mineralization stimulant [165]. Mitsiadis and Pagella et al. [166] confirmed the effects of NGF in the proliferation and differentiation of epithelial and mesenchymal cells, along with accelerated sprouting of nerve fibers in dental tissue during tooth development. Moreover, the level of NGF would increase in pulpitis to accelerate the release of neuropeptides such as SP and CGRP, resulting in pain [167]. Li and Wang [35] delivered a combination of PDGF-BB, NGF, and BDNF into teeth undergoing endodontic treatment, which were then implanted subcutaneously into the dorsum of rats. They found well-vascularized pulp-like tissue formation and positive signals of S-100, indicating that nerve fibers regenerated. Combining chemokines and NGF with cell homing can enhance the regeneration of pulp-like tissue and nerve fibers, providing a good approach for actualizing functional pulp regeneration.

### 5.2. BDNF

BDNF is one of the most investigated and characterized NFs in the nervous system and is also a member of the neurotrophin family. It plays a crucial role in the survival and differentiation of neurons by activating TrkB for the purpose of regulating and maintaining the normal function of the brain. In addition, BDNF can be detected in other tissues and cells—for example, bone, cartilage, tooth germ, heart tissue, and osteoblasts [168]. It was found that BDNF accelerates osteogenesis of BMMSCs through the Erk/Runx2/Osterix pathway to directly regulate endothelial cells’ survival and vascular stability. It also indirectly regulates capillary network formation through VEGF [35,169]. BDNF produced during injury or inflammation may promote axonal growth and peripheral nerve regeneration [170]. 

In dentistry, Nosrat et al. [171] found that the mRNA of BDNF resides mainly in the dental papilla/pulp of postnatal rats using in situ hybridization. They determined that the expression pattern was related to dental innervation. Furthermore, the complement C5a activated by fibroblasts controlled the production of BDNF in pulpitis. Thus, it could promote neuron growth to the injured area [172]. Moreover, de Almeida et al. [131] investigated SCAP co-culture with trigeminal neurons. They found that SCAPs may drive axons through the BDNF signaling pathway to regenerate nerves. Moreover, some studies have found that BDNF and NT-4/5 accelerate the migration of DPSCs in vitro [173]. Luzuriaga et al. [174] demonstrated that BDNF and NT-3 combined with StemPro MSCTM induced the partial reprogramming of ectomesenchymal human DPSCs to generate early neural crest progenitor cells. The capability of BDNF in neuroangiogenesis and the migration of DPSCs suggest its great potential for RET.

### 5.3. NT-3

NT-3, the third member of the neurotrophin family, was discovered by Hohn et al. [175] in 1990. Unlike NGF and BDNF, NT-3 has a higher affinity for TrkC and a lower affinity for TrkA and TrkB [176]. NT-3 has cardiovascular effects, as NT-3- and TrkC-knockout mice had severe heart defects [177,178]. In addition, Cristofaro et al. [178] revealed an angiogenic ability of NT-3 that is associated with TrkC phosphorylation and the phosphatidylinositol 3-kinase–Akt kinase–endothelial nitric oxide synthase pathway. Moreover, it has been found that NT-3 promotes the differentiation of human BMMSCs into osteoblasts in an inflammatory environment [179]. NT-3 is essential in neuronal survival and differentiation. It can promote the reconstruction of neural connections at the lesion site in SCI [180]. Yan et al. [181] cultured BMMSCs with NT-3 and found that the BMMSCs differentiated into neurons. This ameliorated the cognitive function in rats with Alzheimer’s disease. They also proved that the Wnt/β-catechin signaling pathway was involved the process. Moreover, in an SCI rat model, transplanted BMMSCs that overexpressed NT-3 promoted the recovery of motor function and neural regeneration [182]. Additionally, BDNF and NT-3 had a synergistic regulatory effect on the neural differentiation of ADSCs [183]. Given its excellent ability to promote neural regeneration and angiogenesis, NT-3 is an ideal candidate for RET in the future, but more in vivo experiments are necessary.

### 5.4. bFGF

bFGF, or FGF-2, is the earliest FGF to be found in pituitary extracts by Armelin [184] in 1973. As a secretory signal protein, bFGF is expressed in almost all tissues and binds to the tyrosine kinase FGF receptor on the cell membrane. It participates in cell proliferation, differentiation, migration, and angiogenesis. It is related to the activation of the RAS-MAPK, PI3K-AKT, PLC γ, and STAT signaling pathways [185,186]. bFGF is a vital regulator of bone formation and differentiation. After *bFGF* knockout, the bone formation capability of osteoblasts was impaired, and the accumulation of bone marrow fat increased [187]. In addition, bFGF promotes wound healing, accelerates tissue regeneration, participates in neurogenesis, and acts as an angiogenic factor [188]. bFGF increases the size of the neurospheres and the expression of neurogenic markers in DPSCs through intracellular transduction of FGF-FGFR and PLC γ [189]. Lue et al. [190] reported functional recovery in a rat model of SCI after transplanting a hydrogel containing DPSCs and bFGF. Moreover, Liang et al. [191] showed that FGF2-induced human dental pulp cells released NFs to support axon regeneration.

In dentistry, Shimabukuro et al. [192] proved that FGF-2 promotes the proliferation and migration of DPSCs and accelerates the regeneration of the dentin–pulp complex. Local regeneration of the dentin–pulp complex in rats was successfully achieved by using FGF-2, gelatin hydrogel, and a collagen sponge [193]. Additionally, Kim et al. [194] found that FGF-2 enhances the proliferation and migration of stem cells from the inflamed pulp tissue of human functional deciduous teeth (iSHFD), and that ectopic transplantation of iSHFD/FGF-2 increases the formation of dentin-like tissue. Furthermore, Sagomonyants et al. [195] found that bFGF promotes stem cells’ differentiation to odontoblasts by activating the FGFR/MEK/ERK1/2 and BMP/BMPR pathways.

Kim et al. [10] used collagen gel to deliver a variety of GFs—including bFGF—into human teeth, which were then transplanted into rats. They found newborn pulp-like tissue with angiogenesis and neurogenesis. Given the role of bFGF in cell proliferation, angiogenesis differentiation, and neurodifferentiation, as well as its potential for pulp wound healing and regeneration, it can be used as a reserve cytokine for cell homing. Moreover, bFGF has been approved for clinical use by the Japanese Pharmaceuticals and Medical Devices Agency (PMDA) and the United States Food and Drug Administration (FDA). Therefore, bFGF is safe as a candidate for the clinical application of neural regeneration in RET.

### 5.5. IGFBP5

IGF and its six binding proteins (IGFBPs) are crucial for cell growth, proliferation, and differentiation—especially in osteoblasts and fibroblasts [196]. IGFBP5 is related to regulation of the biological function of MSCs, especially in cell growth, differentiation, migration, senescence, and apoptosis [197]. Hao et al. [198] found that IGFBP5 promotes the differentiation of DPSCs into odontoblasts and further enhances dentin formation through the JNK and ErK signaling pathways. Saito and Ohshima et al. [199] reported that IGFBP5 was essential in regulating the survival and apoptosis of DPSCs during tooth development and pulp wound healing. Furthermore, Li et al. [200] found that the overexpression of IGFBP5 upregulates the expression of angiogenic markers (e.g., VEGF, PDGF, angiopoietin-1) in DPSCs, as well as neurogenic markers (e.g., neural cell adhesion molecule, tyrosine hydroxylase, Nestin, β-III-tubulin). These studies confirm the angiogenesis and neurogenic differentiation potential of IGFBP5, but more research is needed to verify its role in neural regeneration.

### 5.6. G-CSF

G-CSF is a glycoprotein belonging to the growth factor cytokine family that is used to clinically treat neutropenia [201,202]. Some investigations have exhibited the neuroprotective ability of G-CSF. In an experimental murine model of cerebral ischemia, G-CSF combined with BMMSCs produced a synergistic effect to promote cell proliferation and differentiation, resulting in early neuronal development and the reduction in cerebral infarction size [203]. Furthermore, G-CSF induces neurogenesis, increases neural plasticity, and reduces apoptosis by activating receptors [204].

In dentistry, G-CSF and dental pulp cells loaded with collagen have been used for pulp regeneration. The results were successful regeneration of pulp-like tissue after 90 days [205,206]. By comparing G-CSF and bFGF in RET, it was found that the two GFs showed similar roles in migration, proliferation, anti-apoptosis, angiogenesis, and neuronal growth in vitro [207]. However, bFGF inhibits mineralization of the DPSCs, while G-CSF promotes mineralization [207]. Meanwhile, G-CSF stimulates the migration and proliferation of endogenous stem cells, similar to SDF-1 [208]. The PMDA and FDA have approved G-CSF for clinical use, and the development of G-CSF in the field of pulp nerve regeneration can be explored further.

## 6. The Role of Scaffolds in Promoting Neural Regeneration

Scaffolds used in RET should not only transport stem cells into the root canal but also regulate the proliferation, differentiation, and metabolism of those stem cells [209]. The properties of biocompatibility, porosity, mechanical strength, and degradation rate should be considered when selecting a scaffold [210]. In addition, the electrical conductivity of the material and its interaction with NFs are also essential when considering the potential for neural regeneration [211].

According to the reports from in vivo and clinical trials showing successful induction of neural regeneration in RET, most used natural biomaterial scaffolds. The most common scaffold materials are autologous platelet concentrates (APCs) and collagen derivatives such as atelocollagen or collagen TE. One study created a unique sandwich structure, composed of hDPSC sheets, TDM/hDPSCs, and Matrigel/hDPSCs. TDM and Matrigel are natural biomaterials [32]. Another clinical study compared the effects of BC, PRF, collagen, and hydroxyapatite on pulp regeneration in vivo. Twelve months later, 11%, 66%, 44.4%, and 33.3% of patients in the BC, PRF, collagen, and hydroxyapatite groups, respectively, were positive to cold tests [29].

The scaffold materials used for RET and neural regeneration can be divided into natural materials, synthetic materials, and composite materials. Since most studies used natural biomaterial scaffolds, the next part of this review focuses on them.

### 6.1. Natural Materials

#### 6.1.1. APC

BC, PRP, and PRF are APCs that are often used for revascularization. BCs are formed by using hand files to mechanically stimulate bleeding at the root tip [212]. PRP and PRF are obtained from peripheral blood by anticoagulation and centrifugation [213]. PRP and PRF are rich in platelets. When platelets are activated, high concentrations of GFs can be produced through degranulation to regulate cells’ chemotaxis, migration, proliferation, and differentiation, thereby promoting tissue repair [214]. A clinical study evaluated radiological results for the root length of 88 immature incisors treated with BC, PRP, PRF, and platelet granules [52]. The study found that the results of all treatment groups were similar and, after an average of 28.25 months, root healing, development, and sensitivity tests showed good results. Various research results shown in Table 2 prove the positive effects of APCs on neural regeneration, since the teeth were positive to sensitivity tests. Although APCs provide necessary GFs for tissue regeneration, further study is needed because of the unpredictability of clot formation, the challenge of obtaining PRP, and the limited curative effect [215].

#### 6.1.2. Collagen 

Collagen is not only the structural protein of human and animal connective tissue, but also the main component of extracellular matrix. The main collagen used for scaffolds is type I collagen. This is the most valuable biomaterial for tissue engineering, drug delivery, and cosmetic surgery [216,217]. Collagen has high biocompatibility and biological activity, and it can promote cell adhesion, migration, and proliferation [218]. Collagen has poor mechanical properties but is adequate for RET, and its mechanical properties can be enhanced through crosslinking with other elements or constructing composite scaffolds [219]. Collagen can carry pulp stem/progenitor cells with or without GFs and anti-inflammatory molecules to form pulp-like tissue in vivo [31,33,35,36,37,38,39,40,41]. After transplanting collagen with CD31^-^ or CD105^+^ pulp cells containing SDF-1 into dogs’ root canals, pulp-like tissue was observed, along with blood vessels and nerve domination [40,41,220]. Collagen hydrogel crosslinked with cinnamaldehyde promoted the proliferation and odontogenic differentiation of human DPSCs [221]. In nerve tissue engineering, collagen can accelerate neuronal growth and neural regeneration, as well as helping to maintain the biological function of neurons [222]. However, the low mechanical strength, high water absorption, and rapid degradation of collagen are not beneficial for neural regeneration [211]. Therefore, collagen often needs further modification for this application. However, these modifications may affect the function of stem cells in pulp regeneration. How to balance the characteristics of collagen with the degree of modification in neural regeneration during RET may be worthy of further investigation.

#### 6.1.3. Polysaccharides

Polysaccharides include HA, alginate, and chitosan. HA is a type of glycosaminoglycan in the extracellular matrix of connective tissue that is composed of repeating disaccharide, β-1, 4-methyl-D-glucuronic acid β-1, and N-methyl-acetyl-D-methyl-D-glucosamine [223]. HA is a powerful inflammatory mediator, and its unique biological and structural characteristics involve it in the regulation of wound repair and morphogenesis [224,225]. It has excellent biocompatibility, low immunogenicity and biological activity. Thus, HA has enormous potential as a scaffold for tissue regeneration [223]. It was found that HA hydrogel containing platelet lysate accelerated mineralized matrix deposition by hDPSCs, providing evidence of pulp regeneration [226]. Yang et al. [227] found that nerve conduits containing HA hydrogel contributed to the morphological and functional recovery of an injured sciatic nerve. 

Chitosan is composed of N-acetyl glucosamine and glucosamine copolymer extracted from chitin [228]. The strengths of chitosan include excellent biocompatibility, biodegradability, low cytotoxicity, and low immunogenicity. It is antibacterial and exhibits outstanding mechanical properties [215,229]. A study investigated DPSCs and GFs combined with chitosan hydrogel and found that they regenerated pulp-dentin-like tissue and promoted root development in dogs [230]. The 3D porous chitosan scaffold constructed by Feng et al. [231] provided a favorable microenvironment for the attachment, survival, and neural differentiation of DPSCs. In addition, Chávez-Delgado et al. [232] successfully used chitosan combined with neurosteroids to regenerate facial nerves in rabbits.

Alginate is the polysaccharide extracted from seaweeds, with covalently linked 1mam 4 and linked β-D mannuronic acid (M) and α-L-guluronic acid (G) units [233]. It is biocompatible, non-cytotoxic, and non-immunogenic, and has been used in diabetes treatment, pancreatic transplantation, and drug/GF delivery systems [234]. One alginate gel containing TGF-β1 promoted odontoblast-like cell differentiation and upregulated dentin matrix secretion [235]. Alginate has insufficient mechanical strength and rapid degradation; thus, it usually needs to be crosslinked with other substances for neural regeneration [236].

#### 6.1.4. Other Biomaterials

Matrigel is a bioactive soluble material derived from the basement membrane of tumor cells [237]. The function of Matrigel is similar to that of the extracellular matrix, and it is usually used as an artificial 3D surface for tissue engineering and differentiation of various cell types [238]. Luzuriaga et al. [239] achieved efficient angiogenesis in human DPSCs cultured with Matrigel. Furthermore, Jeong et al. [240] found that human DPSCs cultured in Matrigel differentiated into odontoblasts and formed pulp-like tissue. In terms of neural regeneration, it has been proven that Matrigel promotes the growth of ADSCs that successfully differentiate into dopaminergic cells [241].

Keratin is a type of natural fibrin associated with epithelial cells and skin appendages that can self-assemble into highly porous fiber scaffolds [218,242]. Gao et al. [243] reported that keratin promoted the proliferation of SCs and the extension of axons. Silk fibroin (SF) is a type of enzymatically degradable material that can be processed into a water-insoluble scaffold with excellent anticoagulant activity, regeneration ability, mechanical strength, elasticity, and a slow degradation rate [244]. An injectable silk fibroin–polydopamine composite hydrogel was fabricated and showed that it could support neuronal growth and promote SCI repair [245].

### 6.2. Synthetic Materials

Compared with natural materials, synthetic materials have better mechanical properties, controllable porosity, thermal stability, and a relatively cheap price. However, they are not as good as natural materials in terms of biocompatibility. This kind of material can be customized to form an ideal structure. The most common synthetic materials used for pulp regeneration are polylactic acid (PLA), poly-L-lactic acid (PLLA), polyepsiloncaprolactone (PCL), polylactic-co-glycolic (PLGA), polyglycolic acid (PGA), polydioxanone scaffolds (PDSs), and polyethylene glycol polylactic (PEG) [210]. Meanwhile, the most common biodegradable artificial materials for nerve regeneration are PGA, PLGA, PCL, and P (DLLA-co-CL) [246].

### 6.3. Composite Scaffolds 

**Figure 2:**
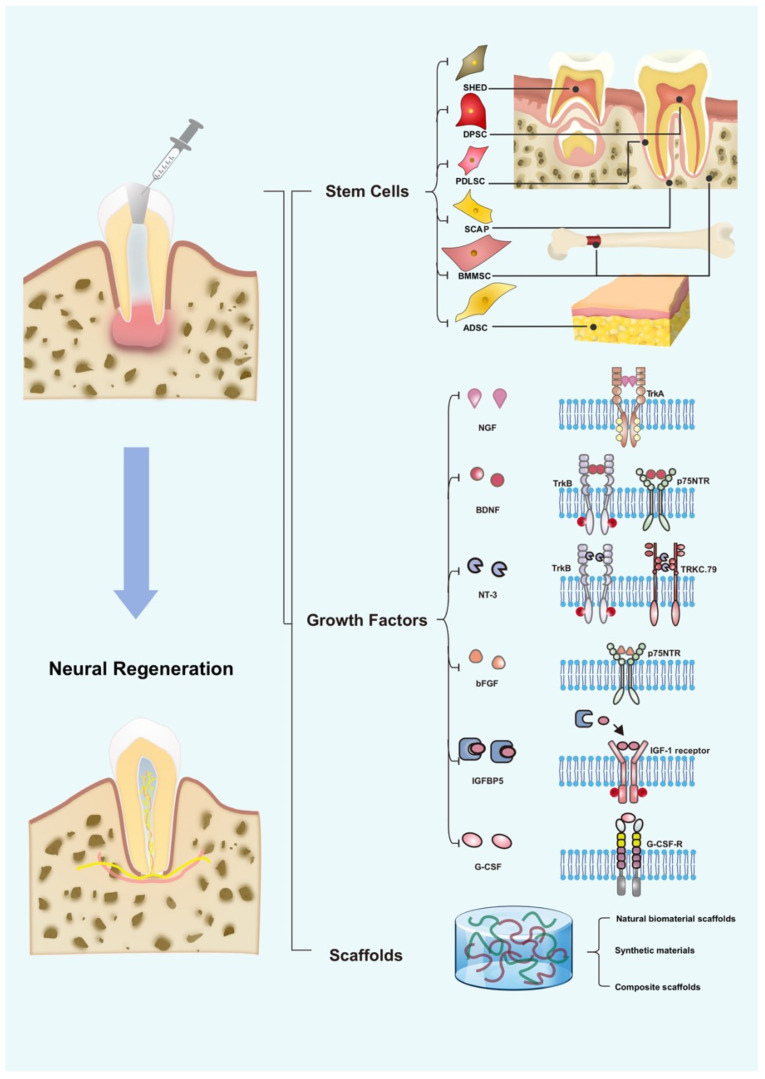
Stem cells, growth factors, and scaffolds in neural regeneration in RET. SHED: stem cell from human exfoliated deciduous teeth; DPSC: dental pulp stem cell; SCAP: apical papilla stem cell; PDLSC: periodontal ligament stem cell; BMMSC: bone marrow mesenchymal stem cell; ADSC: adipose-derived stem cell; NGF: nerve growth factor; BDNF: brain-derived neurotrophic factor; NT-3: neurotrophin-3; bFGF: basic fibroblast growth factor; IGFBP5: insulin-like growth factor-binding protein 5; G-CSF: granulocyte colony-stimulating factor.

Overcoming the shortcomings of single scaffold materials through the combination of two or more materials is a new direction in the development of scaffold materials, such as bioceramic–polymer scaffolds, collagen–HA scaffolds, and collagen–alginate scaffolds (Figure 2). These composites can further promote cell adhesion and tissue/cell–cell interaction, thereby promoting cell proliferation, differentiation, and tissue regeneration [247].

## 7. Interaction between Nerve Regeneration and Maxillofacial Bone Regeneration

The preservation of teeth not only depends on the pulp but is also related to the supporting tissue, including the surrounding alveolar bone. Periapical periodontitis often leads to bone resorption around the apex, and significant bone loss eventually results in tooth extraction. Therefore, promoting the repair of jawbone defects is also key to the treatment of periapical diseases. However, the reconstruction and recovery of bone defects is still a complex matter [248,249,250,251]. An interaction between nerve and bone regeneration has recently been proposed. Brockes et al. [252] found that innervated limbs successfully regenerated while denervated limbs formed stumps after amputation in amphibians. Moreover, denervation before fingertip amputation in mice led to impaired regeneration and significant morphological defects. This emphasizes the neural dependency of bone injury repair and regeneration [253]. 

At present, the relationship between nerve regeneration and mandibular bone regeneration has only been preliminarily discussed. Jones et al. [254] confirmed that SCs’ paracrine signaling pathways are necessary for skeletal stem cells to promote fracture healing by constructing a mandibular denervation model. SCs, derived from the neural crest, are primary glial cells of the peripheral nervous system, which surround axons and promote impulse transmission [255]. Furthermore, SCs secrete paracrine NFs such as BDNF, NGF, and NT-3 to regulate bone regeneration upon bone injury [256,257]. BDNF mediates the effects of osteogenesis–neurogenesis–angiogenesis through the TrkB/ERK1/2 signaling pathway [258]. NGF can not only promote the differentiation, proliferation, and activity of osteoblasts, but also significantly accelerates fracture healing and the rate of bone mineralization [259,260,261]. NT-3 can induce osteogenesis, enhance vascularization, and increase the activity of osteoclasts [262,263]. Therefore, these GFs may be potential candidates to promote both bone regeneration and neural regeneration.

## 8. Limitations and Future Direction

Cell transplantation and cell homing have provided valuable insights into functional pulp regeneration and hold the promise to revolutionize RET and clinical options. However, there are still hurdles to address. First, although various stem cells exhibit the potential to form nerves in vitro and in vivo, the specific mechanism by which stem cells promote neural regeneration is still unknown. Thus, experiments such as lineage tracing and single-cell sequencing should be designed to explore which stem cells dominate neurogenesis during RET. Second, the most commonly used stem cells in transplantation are autologous, avoiding the problem of immunity. However, the in vitro isolation and expansion processes, the cell loss in the storage process, and the high cost are obstacles to clinical applications. It is necessary to find alternative cells and establish stem cell banks. Third, various GFs are added to regulate and promote functional pulp regeneration, and different combinations of factors will have diverse effects. Balancing the series of signals is a complex process. Other molecules that promote neurogenesis are also applicable in RET. For example, exosomes from stem cells are rich in bioactive components and have been proven to improve the repair of peripheral nerve injury [264]. Fourth, the properties of scaffolds for RET and neural regeneration requirements are dissimilar, and no innovative scaffolds have been specifically designed for neural regeneration during RET. Moreover, the scaffold should be compatible with the morphology of the root canal. Three-dimensional (3D) printing seems to be an option, with the potential to build scaffolds with a precise shape and structure to provide a suitable microenvironment for stem cells and GFs. It is expected that a functional pulp regeneration scaffold could be constructed by 3D printing in the near future with ideal biological activity and bionic vascular and nerve effects. 

## 9. Conclusions

The field of RET has certainly made great strides over the course of recent years. Pulp revascularization has achieved satisfactory therapeutic outcomes with the restoration of sensitivity. Stem cell transplantation and cell homing are currently in the preclinical stage. The combination of dental MSCs with neurodifferentiation potential and neurotrophic factors that promote neural regeneration provides excellent conditions for the innervation of newborn dental-pulp-like tissues. Overall, we believe that the ultimate goal of functional pulp regeneration will be achieved.

## Figures and Tables

**Table 1:** Summary of the characteristics of neural regeneration in vivo, including animal and clinical studies of cell homing and stem cell implantation.

Scaffold	Stem Cells	Signal Molecules	Species	Outcome	Reference
(1) Periapical bleeding (2) PRF (3) Collagen (4) Hydroxyapatite	/	/	Human	Radiographically: all succeeded; success rate of cold test: (1) periapical bleeding: 11%; (2) PRF:66%; (3) collagen: 44.4%; (4) hydroxyapatite: 33.3%	Mittal et al., 2021 [29]
/	Human SHED aggregates	/	Pig	Dentin-pulp-like tissue with blood vessels and nerves	Guo et al., 2020 [30]
Atelocollagen	DPSC	G-CSF	Dog	Dentin-pulp-like tissue with blood vessels and nerves	Iohara et al., 2020 [31]
Human treated dentin matrix, and Matrigel	Human DPSC	/	Mice	Dentin-pulp-like tissue with blood vessels and nerves	Meng et al., 2020 [32]
Atelocollagen	DPSC	G-CSF	Dog	Dentin-pulp-like tissue with blood vessels and nerves	Iohara et al., 2018 [33]
/	SHED aggregate	/	Pig; Human	Pig: Dentin-pulp-like tissue with blood vessels and nerves; Human: Pulp-like tissue containing an odontoblast layer, connective tissue, blood vessels, and nerves	Xuan et al., 2018 [34]
Collagen gel	BMMSC	PDGF-BB NGF BDNF	Rat	Well-vascularized pulp-like tissue; positive signals for S-100	Li and Wang, 2016 [35]
Atelocollagen	DPSC BMMSC ADSC	G-CSF	Dog	Dentin-pulp-like tissue with blood vessels and nerves	Murakami et al., 2015 [36]
Atelocollagen	DPSC	G-CSF	Dog	Dentin-pulp-like tissue with blood vessels and nerves	Iohara et al., 2014 [37]
Polymerizing type I collagen hydrogel	Indium-111-oxine -labeled rat pulp cells	/	Rat	Dentin-pulp-like tissue with blood vessels, nerves, and active fibroblastic cells	Souron et al., 2014 [38]
Atelocollagen	DPSC	G-CSF	Dog	Dentin-pulp-like tissue with blood vessels and nerves	Iohara et al., 2013 [39]
Collagen TE	CD31-side population cells from pulp, bone marrow, adipose	SDF-1	Dog	Dentin-pulp-like tissue with blood vessels and nerves	Ishizaka et al., 2012 [40]
Collagen TE	CD105+cells form pulp or adipose	SDF-1	Dog	Dentin-pulp-like tissue with blood vessels and nerves	Iohara et al., 2011 [41]
Collagen	/	bFGF VEGF/PDGF NGF BMP7	Teeth from humans implanted in mice	Dentin-pulp-like tissue with blood vessels and nerves	Kim et al., 2010 [10]

Abbreviations: SDF-1: stromal-cell-derived factor-1; PDGF: platelet-derived growth factor; G-CSF: granulocyte colony-stimulating factor; bFGF: basic fibroblast growth factor; VEGF: vascular endothelial growth factor; BMP7: bone morphogenetic protein 7; BMMSC: bone marrow mesenchymal stem cell; ADSC: adipose-derived stem cell.

**Table 2:** Summary of the characteristics of neural regeneration in clinical revascularization.

Revascularization Treatment	Characteristics of Samples	Radiological or Pathological Results	Evidence of Successful Neural Regeneration	Reference
BC	Immature permanent teeth of 40 patients	Increased root length and thickness	The EPT on the sixth week demonstrated a positive response	Sajjad et al., 2022 [42]
BC PRF	Mature anterior teeth of 20 patients	Periradicular healing in both groups	Different readings of tooth sensitivity between preoperative, 6 months, and 12 months	Youssef et al., 2022 [43]
BC	23 immature necrotic permanent teeth	Increased root length and thickness	53.8% and 50% responded to EPT after 3 and 8 years, respectively	Abu Zeid et al., 2021 [44]
Plasma-derived biomaterial with human umbilical cord MSCs	Mature anterior teeth of 18 patients	After 12 months, the treatment showed 100% clinical efficacy	Increased positive pulp response at 12-month follow-up	Brizuela et al., 2020 [45]
BC	51 immature permanent teeth with pulp necrosis	91.4% showed root development.	54% responded positively to cold or EPT, and 30% responded to both tests	Chrepa et al., 2020 [46]
BC	16 traumatized permanent incisors with open apices	56.3% root resorptions, 31.3% ankylosis, and 92.9% discolorations	81.3% of teeth regained sensitivity	Mittmann et al., 2020 [47]
BC PRF	The upper front teeth of an 11-year-old child	Increased root length and thickness; closure of apex	After 12 months, both teeth were positive to the cold test and EPT	Nagaveni et al., 2020) [48]
BC	Traumatized upper immature anterior teeth of 15 children	Periapical healing, root walls thickened, and apical closure closed	One tooth was positive to the cold test; four teeth were positive to EPT	Nazzal et al., 2020 [49]
BC	28 mature teeth	92.3% favorable clinical and radiographic outcomes	Half of the teeth responded to the EPT	Arslan et al., 2019 [50]
MSCs PRF	Tooth #28 of a 50-year-old man	Normal under percussion and palpation	Delayed response to cold test; positive to EPT	Meza et al., 2019 [51]
BC PRP PRF PP	88 immature incisors of 67 children	86 teeth showed periapical healing and root development	86 teeth were positive to sensitivity tests	Ulusoy et al., 2019 [52]
BC PRF	Five patients per group	Increased root length and thickness; closure of apex	Positive to sensitivity test between 6 and 9 months of follow-up	Lv et al., 2018 [53]
BC	An immature permanent tooth	Increased root length and thickness, and complete osseous healing	Positive to cold test	Mustafa, 2018 [54]
PRF	15 patients with mature necrotic pulp	Complete healing of periapical lesion	Different readings of tooth sensitivity between preoperative and 12 months	Nageh et al., 2018 [55]
BC	Immature maxillary incisors of 15 children	Apical foramen narrowed	One-third of the teeth were responsive to EPT	Nazzal et al., 2018 [56]
BC	15 patients with immature and mature permanent teeth	10/13 root development and apical closure	2/13 were positive to EPT	Neelamurthy et al., 2018 [57]
PRP BC	30 non-vital immature permanent teeth	Lesion size decreased; bone density increased; continued root development	After 5 months, sensitivity tests elicited a delayed positive response in 23 sites	Alagl et al., 2017 [58]
Experimental: BC + Bio-Gide collagen membrane Control: BC	46 non-vital immature teeth	Thickening of the dentin wall	EPT was achieved in 18% of the control group and 33% of the experimental group	Jiang et al., 2017 [59]
BC	20 teeth with dens evaginatus treated	Apical diameter and root length increased	Five teeth responded to the pulp sensitivity test during the 1-year follow-up period	Li et al., 2017 [60]
PRF PRP BC	60 patients with immature permanent teeth	Increased root length and thickness; healing of periapical wound	15% in PRF, 13.30% in BC, and 15.8% in PRP were positive to the vitality test	Shivashankar et al., 2017 [61]
BC	4 infected necrotic primary second molars	At six months, complete periradicular healing and remained symptomless	Positive to cold test	Ulusoy and Cehreli, 2017 [62]
BC	Maxillary left central incisor of an 8-year-old child	Increased root length and thickness; healing of periapical wound; closure of apex	Responded to cold and EPT as normal teeth	Farhad et al., 2016 [63]
BC	#45 of a 10-year-old girl	Pulp-like tissue with blood vessels	The neurovascular bundles in the pulp-like tissue were NF (+)	Meschi et al., 2016 [24]
PRF	Immature permanent tooth of an 11-year-old boy	Increased root length and thickness; healing of periapical wound; closure of apex	Positive to cold test and EPT similar to adjacent teeth after 3 months	Nagaveni et al., 2016 [64]
PRP	An avulsed mature incisor of an 11-year-old boy	Resolution of periapical radiolucency	Positive to thermal test and EPT	Priya et al., 2016 [65]
PRF	A traumatized, necrotic, immature tooth	Increase in root length and an intact lamina dura	Remained negative to cold test but positive to EPT at 24 and 36 months	Ray et al., 2016 [66]
PRF	Left posterior tooth of a 13-year-old male	Increased root length and thickness; resolution of periapical wound; closure of apex	Positive to cold test and EPT	Subash et al., 2016 [67]
PRP BC	20 immature teeth	Complete apical closure and increased root length	5 teeth in PRP and 2 teeth in BC were positive to vitality test	Bezgin et al., 2015 [68]
BC	4 mature teeth	Root formation increased and dentin wall thickened	3/4 teeth responded positively to sensitivity test.	Dudeja et al., 2015 [69]
BC	An immature permanent tooth	Increased root length and thickness; closure of apex	Neurons and nerve fibers were observed	Lei et al., 2015 [70]
PRF	An immature permanent tooth	Increased root length and thickness; closure of apex	Positive to cold test and EPT	Nagaveni et al., 2015 [71]
PRP	Maxillary left lateral incisor of a 16-year-old male	Increased root length and thickness; resolution of periapical wound; closure of apex	Not response to cold tests; delayed positive response to EPT	Sachdeva et al., 2015 [72]
BC	3 premolars and 13 incisors	Incomplete apical closure: 47.2%; complete apical closure: 19.4%. Change in root length varying from −2.7% to 25.3%, and change in root dentin thickness ranging from −1.9% to 72.6%	5/16 teeth were positive to EPT at 18-month recall	Kahler et al., 2014 [73]
BC	3 immature permanent central incisors	Complete root development	Positive to pulp test	Farsi et al., 2013 [74]
PRF	Immature right maxillary central incisor of a child	Increased root length and thickness; closure of apex	Positive to cold test and EPT	Keswani and Pandey, 2013 [75]
PRF	Tooth #21 of an 11-year-old child	Resolution of periapical rarefaction, further root development, and apical closure of the tooth	Positive to EPT and cold test	Mishra et al., 2013 [76]
BC	Central incisors of an 8.5-year-old boy	Increased root length and thickness; closure of apex	Positive to cold tests at 12 months and EPT at 18 months	Cehreli et al., 2012 [77]
BC	An immature permanent incisor	Root development and apical closure	Positive to sensitivity tests at 1–3-months	Miller et al., 2012) [78]
PRF	Immature incisor of a 9-year-old child	Increased root length and thickness; resolution of periapical wound; closure of apex	Positive to cold test and EPT	Shivashankar et al., 2012 [79]
BC	6 immature permanent molars of children	Increased root length and thickness; resolution of periapical wound; closure of apex	Two teeth were positive to cold test	Cehreli et al., 2011 [80]
BC	An immature permanent incisor of a child	Root development and apical closure	Positive to EPT	Iwaya et al., 2011 [81]
PRP	Maxillary premolar tooth of a child	Increased root length and thickness; resolution of periapical wound; closure of apex	Positive to cold test and EPT	Torabinejad and Turman, 2011 [82]
BC	6 immature teeth with apical periodontitis	Resolution of periapical lesions; continued root development	Two teeth positive to vitality test	Petrino et al., 2010 [83]
BC	A mandibular premolar	Increased root length and thickness; resolution of periapical wound; closure of apex	Positive to sensibility test	Thomson and Kahler, 2010 [84]
BC	Immature permanent teeth of 12 patients	Increased root thickness; closure of apex	3 teeth positive to pulp test	Ding et al., 2009 [85]
BC	2 bilateral mandibular premolars with dens evaginatus of an 11-year-old girl	Increased root length and thickness; resolution of periapical wound; closure of apex	Positive to cold test	Reynolds et al., 2009 [86]
BC	Lower-right second premolar of a child	Increased root thickness; closure of apex	Positive to cold test	Banchs and Trope, 2004 [87]
BC	An immature premolar of a 13-year-old patient	Increased root thickness; closure of apex	Positive to EPT	Iwaya et al., 2001 [88]

Abbreviation: EPT: electric pulp test; BC: blood clot; PRF: platelet-rich fibrin; PP: platelet pellet; PRP: platelet-rich plasma.

## Data Availability

Not applicable.
